# Quality of the Healthcare Services During COVID-19 Pandemic in Selected European Countries

**DOI:** 10.3389/fpubh.2022.870314

**Published:** 2022-05-12

**Authors:** Magdalena Tuczyńska, Rafał Staszewski, Maja Matthews-Kozanecka, Agnieszka Żok, Ewa Baum

**Affiliations:** ^1^Students Scientific Circle of Maxillofacial Orthopaedics and Orthodontics, Poznan University of Medical Sciences, Poznań, Poland; ^2^Department of Hypertension, Angiology and Internal Medicine, Poznan University of Medical Sciences, Poznań, Poland; ^3^Department of Social Sciences and the Humanities, Poznan University of Medical Sciences, Poznań, Poland; ^4^Division of Philosophy of Medicine and Bioethics, Poznan University of Medical Sciences, Poznań, Poland

**Keywords:** COVID-19, healthcare, quality, Europe, economy

## Abstract

**Background:**

There are several definitions of the quality of healthcare services. It may be defined as a level of value provided by any health care resource, as determined by some measurement. Scientists use a variety of quality measures to attempt to determine health care quality. They use special indicators or based on a patients' or healthcare professional's perception. This article aims to provide a short review of the available data on the quality of healthcare services in selected European countries during the COVID-19 pandemic.

**Methodology:**

The research was done by the use of online databases such as PubMed, Google Scholar, and Science Direct. All the studies focused on the quality of healthcare services, yet the studies used different methods to measure this quality. In addition, the results of the authors' survey on the assessment of the quality of healthcare services before and during the COVID-19 pandemic were presented.

**Results:**

Among twelve studies, four were from the United Kingdom and one each of Catalonia, Italy, Sweden, Poland, Netherlands, France, Germany, Belgium. Patients in the United Kingdom felt that the quality of services was good during the pandemic, whereas the quality declined in the other studies cited. The results of our research also revealed a decrease in the quality of healthcare services provided.

**Conclusions:**

Nevertheless the development of telemedicine has had a positive impact on the quality of healthcare services. The COVID-19 pandemic has undoubtedly affected most European countries' quality of healthcare services.

## Introduction

Quality of healthcare is a theoretical concept and is therefore difficult to measure directly. There is no single definition for quality of healthcare. Different institutions and people often mean other things when using it. One definition states that it is the degree to which health services for individuals and populations are effective, safe and people-centered (1). According to the World Health Organization (WHO), quality of care is defined as the degree to which health services for individuals and populations increase the likelihood of desired health outcomes (2). One way to measure quality is routine data which is a readily available and potentially rich source of information about large numbers of patients. Unfortunately, this method has its limitations: the clouding effects of chance and sometimes precarious nature of the underlying data (3). Another way is to use quality indicators, sometimes called measures, which should provide information about a quality goal (a clear statement about the intended purpose or objective), a measurement concept (a specified method for data collection and calculation of the indicator) and an appraisal concept (a description of how a measure is expected to be used to judge quality) (1). Quality can also be measured by healthcare professionals or patients' perceptions. Some authors say that patient perceptions of quality are meaningful and should be a primary focus of attention within the healthcare system. Still, there is a concern that patients' do not know enough (or perhaps even care enough) about medicine to have perceptions of healthcare quality that should be taken seriously by the delivery system (4).

Since, World Health Organization (WHO) declared a pandemic of the SARS novel coronavirus-2 (SARS-CoV-2), responsible for coronavirus disease 2019 (COVID-19), health systems, hospitals and units of care has been assessed for the risks of outbreaks of new emerging infectious diseases as well as biological, and climate risks (5). Most European countries take actions to expand their healthcare workforces as the COVID-19 pandemic developed included an invitation for recently retired staff back into general medical practice and arranging for near-qualified students to start working in the health service (6). A rapid and unexpected COVID-19 pandemic outbreak has led to a breakdown of the health systems in the world. This has also led to a decrease in the quality of health care. Hospital wards and intensive care units were overwhelmed (7). Some hospitals have decided to reschedule non-urgent visits to ensure patients' and personnel safety (8), or patients have canceled medical appointments by themselves for fear of infection (9). The spread of the SARS-CoV-2 virus has had a global impact on the world economy and access to and quality of healthcare services (10).

This article aims to provide a short review of the available data on the quality of healthcare services in selected European countries during the COVID-19 pandemic and present the results of the author's questionnaire on the quality of health services in Poland.

## Materials and Methods

The authors of this article searched the PubMed, Google Scholar, ScienceDirect databases between December 2021 and January 2022. Data were screened using MeSH (Medical Subject Headings) terms: “Healthcare Quality”, “Assessment of Healthcare Quality”, “Healthcare Quality, Access and Evaluation”. They also added the terms: “COVID-19”, “pandemic”, and “SARS-CoV-2”. The authors chose the mini-review format due to the narrow scope of the inclusion criteria. The investigation included studies focused on quality of healthcare services in Europe. It was important during screening process to include research-based data from different countries. Studies which met criteria: studies on quality of healthcare services during pandemic, studies conducted among European patients, studies in all languages were included. Studies that did not meet the criteria were excluded. The studies which met the inclusion criteria were listed and further reviewed. In case of bias the second author was the decisive person. In addition, the results of a survey on the assessment of the quality of healthcare services before and during the COVID-19 pandemic among 256 patients from Poland are presented.

## Results

Twelve publications from the examined literature were found for this mini-review. From each of the included studies, the following data were extracted: author, year, country, the scope of health services, study design and methodology and outcome (Table 1). Among the twelve studies, four were from United Kingdom (*n* = 4) and one each of Catalonia (*n* = 1), Italy (*n* = 1), Sweden (*n* = 1), Poland (*n* = 1), Netherlands (*n* = 1), France (*n* = 1), Germany (*n* = 1), Belgium (*n* = 1). All the studies focused on the quality of healthcare services, yet the studies used different methods to measure this quality.

**Table 1:** An mini overview of studies' outcomes conducted in Europe regarding to quality of healthcare during COVID-19.

**References**	**Country**	**Scope of health services**	**Study design and methodology**	**Outcome**
Coma et al. (11)	Catalonia	Primary care	A retrospective descriptive study was conducted in the 288 primary care practices (PCP) of the Institut Català de la Salut. The study period was the first 4 months of 2019 and 2020. For this study, 34 quality indicators of different types were included: adequacy of treatment (4 indicators), follow-up of chronic diseases (5), control of chronic diseases (10), screening (7), vaccinations (4) and quaternary prevention (4).	A negative effect was observed on 85% of the quality indicators in March and 68% in April. 90% of the control indicators had a negative impact, highlighting the control of LDL cholesterol and blood pressure control. The indicators with the most significant negative effect were screening, such as the indicator of diabetic foot screening.
Fieux et al. (12)	France	Otolaryngology—telemedicine	A prospective study was performed in the otolaryngology department of a university hospital center. A satisfaction survey was carried out over a 7-day inclusion period during lockdown among 100 patients. The questionnaire consisted of 12 questions.	Overall satisfaction was 87%. The sound quality was judged poorly or unsatisfactory by 24% of patients and video quality by 39%. On the other hand, 94% of patients agreed or ultimately agreed that communication was accessible.
Danhieux et al., 2020 (13)	Belgium	Chronic care	A qualitative study was conducted in 16 primary care practice among twenty-one people (doctors, nurses, dieticians) who were interviewed, using semi-structured video interviews.	Changes in organization with a collective shift toward COVID-19 care, and reduction of chronic care activities, less consultations, and staff responsible for self-management support put on hold, in ensuring quality chronic care were observed.
de Joode et al. (14)	Netherlands	Oncology	The patients' perspective on oncology care was investigated using an online survey, consisted of 20 questions between March 29th 2020 and April 18th 2020. Five thousand three hundred two patients with cancer completed the survey.	Patients with delay (55%) and discontinuation (63%) of treatment, were very concerned about these consequences of the COVID-19 pandemic.
Brislane et al., 2021 (15)	United Kingdom, Ireland	Obstetric care	Between May 3rd and June 28th, 2020, 314 women from the United Kingdom and 23 from Ireland validated the questionnaire to quantify healthcare quality using the 46-item Quality of Prenatal Care Questionnaire (QPCQ).	72% of women report good quality of care.
Golinelli et al. (16)	Italy	Orthopedic	The retrospective cohort study included 5,379 patients with hip fractures. Surgery rate, surgery timeliness, length of hospital stay, timely rehabilitation, and 30-day mortality for each patient were analyzed. Data was evaluated monthly (2020 vs. 2019).	There was a significant increase in the proportion of patients that did not undergo timely surgery and a substantial increase in mortality.
Key et al., 2021 (17)	United Kingdom	General Health	A single-center patient experience survey was conducted among 704 patients across the Cardiff and Vale University Health Board. The quality of care was assessed on a scale of 1 to 10.	The mean score for quality of care was 9 (with ten being the highest). The majority of patients reported that they believed adequate staff in the hospital to care for them (66% always, 21% often).
Kludacz-Alessandri et al. (18)	Poland	Primary care—telemedicine	The data was collected during two sessions: on February 25th 2021–February 26th 2021 and March 11th 2021–March 12th 2021 through an anonymous questionnaire assessing the quality of primary medical care among 98 patients.	Patients rated primary healthcare services during the COVID-19 pandemic through telemedicine quite highly.
Lakshin et al. (19)	Germany	Pediatric surgery—telemedicine	A cross-sectional analysis using three surveys between 6/2020 and 10/2020 was conducted based on anonymous survey among 81 pediatric's surgeons and 86 families with telemedical appointments at the Department of Pediatric Surgery of the University Hospital of Frankfurt.	91% of the surgeons providing telephone visits think that patients are satisfied with the service, 89% of those with video visits, and the rest could not tell; 96% of the patients found the connection quality during their telephone consultations sufficient. 97% experienced no technical problems during the call. When asked to compare a telemedical visit to a traditional, in-person one, 33% found it inferior, 44% found it to be equal, 4% said it was superior while 19% could not tell.
Douiri et al. (20)	United Kingdom	Stroke care	A registry-based cohort study of patients with acute stroke admitted to hospitals in England, Wales, and Northern Ireland between October 1st, 2019, and April 30th, 2020, and equivalent periods in the three prior years.	Care quality was maintained or improved for all care quality (e.g., Brain scan within 1 h; swallow screen within 4 h; direct admission to Stroke Unit within 4 h; stroke specialist physician assessment within 24 h).
Nymark et al. (21)	Sweden	Cardiology	The MISSCARE Survey-Swedish version was conducted among 43 registered nurses and nurse assistants at a cardiology department. The data were compared with a reference sample–59 registered nurses and nurse assistants at a cardiology department who filled a baseline survey conducted in October 2019.	Significant differences were found between the COVID-19 and the reference sample concerning the perception of patient safety and quality of care. The nursing staff in the COVID-19 sample perceived the quality of care to be lower than those in the reference sample (85.7% vs. 98.3%, *p* = 0.04).
Kanavaki et al. (22)	United Kingdom	Nephrology	The observational study using mixed-methods study involving kidney patients, their significant others and 8 Healthcare Professionals (HCP) conducted by psychologist.	Most participants found the challenges of remote patient assessment and monitoring unsatisfactory; there were delays in patients receiving appropriate treatment leading to sub-optimal care; more than one in three participants described difficulties in HCP-patient communication.

### Quality Assessment of In-person Healthcare Services

A study based on the Quality of Prenatal Care Questionnaire (QPCQ), conducted in the United Kingdom among pregnant and postpartum women, showed that 72% of them reported good care quality. Quality of care was significantly correlated with birth partners' permission to attend birth, whereby those permitted to be accompanied, reported good quality of care (76 ± 14%) compared to those not allowed (63± 10%) (15). Other studies from the United Kingdom also provided information on the good quality of healthcare during the COVID-19 pandemic. Respondents of a single-center patient experience survey scored the quality 9 of 10, with 10 being the highest score (17), and cohort study of patients with acute stroke revealed that care quality was maintained or improved for all care quality (e.g., brain scan within 1 h; swallow screen within 4 h; direct admission to Stroke Unit within 4 h; stroke specialist physician assessment within 24 h) (20). However, based on patient and healthcare professionals' (HCPs) interviews, it was found that the quality of health services was not satisfactory. More than one in three participants described difficulties in HCP-patient communication, there were also delays in patients receiving appropriate treatment leading to sub-optimal care (22).

Different results were obtained in studies from other European countries. Retrospective descriptive study conducted in Catalonia that based on the 34 quality indicators (adequacy of treatment–4 indicators, follow-up of chronic diseases–5, control of chronic diseases–10, screening–7, vaccinations–4 and quaternary prevention–4) revealed that in the year 2020 a negative effect on 85% of the quality indicators in March and 68% in April was observed. Additionally, the indicators with the most significant negative impact were those of screening, such as the indicator of diabetic foot screening (11). Then a survey study among registered nurses and nurse assistants at a cardiology department in Sweden reports that the nursing staff in the COVID-19 pandemic perceived the quality of care to be lower than before it started (21). Overall, 75% of oncology patients from the Netherlands stated in an online survey that the COVID-19 pandemic did not influence their contact with the hospital. However, more than half of patients–55% with delayed treatment and 63% with interrupted treatment, declared anxiety about the consequences of pandemic COVID-19 (14). Further, a retrospective cohort study conducted in Italy showed a significant increase in the proportion of patients that did not undergo orthopedic surgery on time (16). Changes in organization with a collective shift toward COVID-19 care, and reduction of chronic care activities in ensuring quality chronic care were observed in Belgium among chronic care professionals. Additonally, fewer consultations, and staff responsible for self-management support put on hold were shown (13).

### Quality Assessment of Healthcare Services *via* Telemedicine

The use of telemedicine during the COVID-19 pandemic seems to be a suitable method. The study conducted in Poland through an anonymous questionnaire assessing the quality of primary medical care showed that patients rated primary healthcare services during the COVID-19 pandemic through telemedicine quite highly. Unfortunately, some patients have a problem making an appointment or booking it with a general practitioner of their choice. Additionally, almost half of respondents reported having trouble contacting the Health Care facility *via* telephone or Internet (18). These findings are also confirmed by the study conducted in France among patients in the otolaryngology department. Although 87% of respondents express overall satisfaction with medical services *via* telemedicine, some judged not satisfactory sound or video quality. Many as 49% of patients believe that teleconsultation was not equivalent to in-person appointments (12). Similar results to those mentioned above were obtained by researchers from Germany among pediatric surgeons and patients. Most of the surgeons stated that their patients were satisfied with telephone (91% respondents) and video (89% respondents) visits. Almost all patients experienced no technical problems during the call and found the connection quality during their telephone consultations sufficient. When asked to compare a telemedical visit to a traditional, in-person one, 33% found it inferior, 44% found it to be equal, 4% said it was superior while 19% could not tell (19).

### Outcomes of Authors' Questionnaire

**Figure 1:**
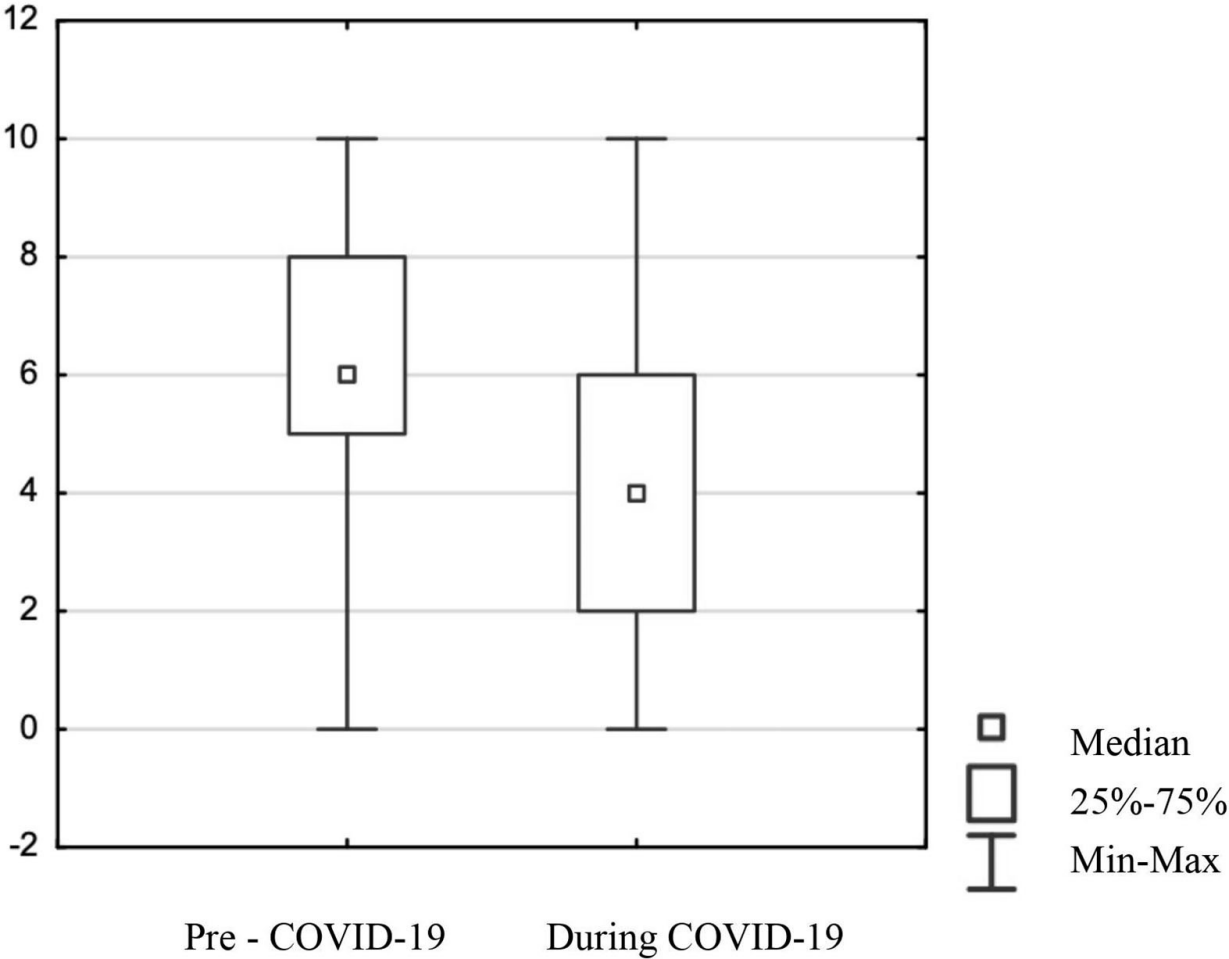
Comparison of respondents' assessment of the quality of healthcare services in Poland before and during the COVID-19 pandemic.

The study was conducted among 265 adult participants living in Poland, of whom 181 were women, 82 were men, and two did not define sex. Most respondents were under 60 for women and under 65 for men. The study was conducted using the online survey prepared in Polish, which is the native language of the respondents, and it contained questions: ‘How would you rate the quality of healthcare services before and during the COVID-19 pandemic?' Respondents were asked to mark their replies on a visual analog scale (VAS) of 1–10, where one stood for very bad and 10 for very good. The analysis of the change in the quality of medical services due to the COVID-19 pandemic showed statistically significant data. The results revealed that the mean score for quality healthcare services before the COVID-19 pandemic in Poland was “6” (Mean score–6.22 and Standard Deviation–2.14) and during was only “4” (Mean score–4.39 and Standard Deviation–2.73) in the group of all respondents (Figure 1). Comparison of changes in ratings of healthcare quality by gender revealed no differences in ratings of quality. Further questions concerned the use of medical services during the COVID-19 pandemic and what type of services were used (government-funded, private sector, primary care, specialist services). Nearly 80% of respondents utilized medical services during the COVID-19 pandemic. Utilization of state-funded services was declared by 75% of respondents and from the private sector by 57%. Both primary healthcare and specialist medical services were declared to be used by a similar number of respondents i.e., 67%. When it comes to responses in the group of women only, the values were as follows: 86% of women utilized medical services during the COVID-19 pandemic, 76% utilized state-funded and 63% private sector healthcare services, only 35% of women utilized primary healthcare services and as many as 72% specialist medical services. Whereas, in the group of men 68% utilized medical services, 70% utilized state-funded services, 39% private sector services, 29% primary healthcare services, and 57% specialist medical services. Based on the Pearson Chi2 test, statistically, significant differences were found only for gender in relation to utilization of medical services (*p* = 0.00107), to private sector services (*p* = 0.00193), to specialist medical services (*p* = 0.02018). This means that women were more likely than men to utilize medical services both private and specialist. For these variables, an adjustment of the original analyses was performed using a multiple regression model to compare changes in quality ratings relative to the use of healthcare services, relative to use of private-sector health services, relative to use of specialized healthcare services by gender. Adjustments to the original analyses were made only for variables used in the analyses that showed gender dependence in the first place. Multiple regression analysis showed that in none of the above cases did the variable “gender” have an association between change in quality rating and utilization of health services, private sector services, and specialized services.

These answers showed that respondents think that Poland's quality of health services has declined due to the pandemic, which confirms findings from most other European countries. Even before the pandemic, health services in Poland were underfunded by the government, resulting in long waiting times for appointments, difficulties in making specialist appointments, and difficulties in obtaining prescriptions. The outbreak of the COVID-19 pandemic has only exacerbated the problem. As the survey results show the quality rating before the COVID-19 pandemic was not too high, and it worsened during the pandemic according to patients. Such results should draw the attention of those in power to increase the quality of healthcare services regardless of the pandemic.

## Discussion

The aim of the study was to provide a mini review of the available data on the quality of healthcare services in selected European countries during the COVID-19 pandemic and present the results of the author's questionnaire on the quality of healthcare services in Poland. Over the past decade, spending on healthcare services in most European countries has shown only slight growth. In the EU, almost one-third of public healthcare expenditure is used to cover the running expenses of inpatient curative institutions. Over the years, hospitals have been subject to increasing pressure and have often been seen as a major potential source for cuts in public health systems. Data from the World Health Organization (WHO) show that since the beginning of the 1990s, the number of hospitals has been drastically reduced throughout Europe, but particularly in Belgium and Italy. Underfunding health services can affect the quality assessment (23). However, studies showed the high quality of healthcare services in the United Kingdom. It can be explained by the protocols implemented by the government. Patients were encouraged to register online or by phone, then a teleconsultation was used to determine if the patient required a face-to-face visit. If they did require a face-to-face visit, it was provided later that day but the aim was to deal with as many queries as possible by telephone or a video call. The implementation of home visits in some areas of England has also proved to be a good solution, especially for patients who would find it difficult to travel. It seems that the key to good quality health services appears to be the UK Government's commitment to financially support general practices (24). Other countries have also implemented improvements for patients in times of the COVID-19 pandemic. In Poland, the government has launched a mobile application called STOP COVID-ProteGO Safe. The application allows patients to track potential contact with someone infected with the SARS-CoV-2 virus, to obtain a referral for a COVID-19 test, or to read recommendations on how to react when the user is feeling unwell (advice, phone numbers, etc.) (25). On the other hand, an interview-based study of nephrology patients and healthcare professionals in the United Kingdom found that during the COVID-19 pandemic, communication was difficult, remote assessment and monitoring of patients proved unsatisfactory, and patients received appropriate treatment with a delay. These reports are particularly noteworthy because studies have shown that kidney disease affects the quality of life, activity, and mental state of the patients (26). Very often chronic kidney disease co-exists with other diseases, such as diabetes, which only adds to the difficulties of treatment. Therefore, it can be concluded that if the quality of healthcare services declines, the quality of life of nephrology patients also suffers (27). High-quality healthcare is particularly important among older people, who are more likely to present chronic diseases. It was noted that older people requiring assistance with activities of daily living had a three times higher risk of mortality from COVID-19 than those who were independent. Increased risk of COVID-19 among geriatric patients should be included in guidelines developed by public health services (28–30). Furthermore, there was also a noticeable decline in the quality of healthcare services among women during the COVID-19 pandemic. Although referring to the results of our questionnaire, there were no statistically significant differences between women and men in the assessment of the quality of healthcare services in Poland, it is worth mentioning that the restrictions implemented by the government had significant consequences on medical services provided to women. General women's prophylactic healthcare programs were suspended or delayed. In addition, difficult access to specialists, lack of solutions for women in quarantine or isolation during the onset of the pandemic condition, restriction of the possibility for a relative to attend the consultation and delivery, separation of mothers and newborns were observed (31).

## Conclusion

The pandemic caused by the SARS-CoV-2 virus has undoubtedly affected most European countries' quality of healthcare services. Curiously, the study reports carried out in the United Kingdom differ from this. Patients in the United Kingdom felt that the quality of services was good during the pandemic, whereas, the quality declined in the other studies cited. Our online survey also confirmed this decline. In addition, one of the few benefits of the pandemic was the development of telemedicine. Studies indicate that the quality of medical services *via* telephone or the Internet has been good, but it should not be forgotten that it also has its drawbacks.

## Author Contributions

MT and RS designed the concept, did the literature search and wrote the manuscript. MM-K and AŻ helped in the literature search. EB was the decision-maker in case of bias and edited and revised the manuscript. MT, MM-K, and EB designed authors' own questionnaire, conducted the survey and collected the results. All authors contributed to the article and approved the submitted version.

## Conflict of Interest

The authors declare that the research was conducted in the absence of any commercial or financial relationships that could be construed as a potential conflict of interest.

## Publisher's Note

All claims expressed in this article are solely those of the authors and do not necessarily represent those of their affiliated organizations, or those of the publisher, the editors and the reviewers. Any product that may be evaluated in this article, or claim that may be made by its manufacturer, is not guaranteed or endorsed by the publisher.
